# Biosynthesis-Informed Putative Annotation Reveals Alkaloid Profiles Across Tissues of *Anisodus luridus*

**DOI:** 10.3390/metabo16090677

**Published:** 2026-09-14

**Authors:** Fangyu Zhao, Yuanjiang Xu, Xiaozhong Lan, Zhen Li

**Affiliations:** 1 State Key Laboratory of Plant Environmental Resilience, College of Biological Sciences, China Agricultural University, Beijing 100193, China; zhofangyu150@163.com; 2 Key Laboratory of Xizang’s Medicine Resources Conservation and Utilization of Xizang Autonomous Region, Xizang Agriculture and Animal Husbandry University, Linzhi 860000, China; 3 Chongqing Institute of Traditional Chinese Medicine, Chongqing 400065, China; yuanjiangxu@126.com

**Keywords:** *Anisodus luridus*, tropane alkaloids, phenylpropanoid polyamine alkaloids, metabolomics, tissue-associated distribution, UPLC-HRMS

## Abstract

**Background/Objectives**: Tropane alkaloids (TAs) and phenylpropanoid polyamine alkaloids (PPAs) are major alkaloid constituents of solanaceous plants; however, the tissue-specific distribution of these compounds in *Anisodus luridus*, a medicinal plant native to southeastern Xizang, remains incompletely characterized. **Methods**: A biosynthesis-informed putative annotation workflow was developed using ultra-performance liquid chromatography–high-resolution mass spectrometry (UPLC-HRMS) to characterize alkaloid profiles across seven tissues (root, stem, leaf, calyx, corolla, ovary, and seed). Putative annotations were guided by substrate–product structural relationships, predicted enzyme-catalyzed modifications, and diagnostic tandem mass spectrometry (MS/MS) fragmentation patterns. **Results**: Eighteen alkaloid features were characterized, comprising 6 TAs and 12 PPAs. Hyoscyamine and scopolamine were identified using authentic standards, and the remaining 16 features were putatively annotated, including dihydroanisodamine, hexosylhyoscyamine, and several PPAs not previously reported in *A. luridus*. TAs were predominantly enriched in roots and ovaries, whereas PPAs accumulated in floral tissues and leaves. Orthogonal partial least squares discriminant analysis (OPLS-DA) identified scopolamine (variable importance in projection (VIP) = 1.30) as a principal variable associated with separation among tissue metabolic profiles. **Conclusions**: This workflow supports the putative annotation of alkaloids in non-model medicinal plants. The observed tissue-specific distribution patterns provide a basis for future comparative studies with *A. belladonna* and for evaluating cultivation, tissue-selective harvesting, and extraction strategies.

## 1. Introduction

Tropane alkaloids (TAs), including hyoscyamine and scopolamine, are bioactive specialized metabolites widely represented in the Solanaceae. Their anticholinergic activity underpins their clinical use as antispasmodic and analgesic agents and contributes to sustained global demand [1]. *Atropa belladonna* is currently a principal industrial source of TAs, and its dried aerial parts are listed in the pharmacopoeias of several countries [2]. However, the total TA content of *A. belladonna* ranges from 0.2% to 2%, and in planta conversion of hyoscyamine to scopolamine is limited [3]. Geographical and climatic constraints increase extraction costs and limit production capacity, contributing to persistent supply constraints [4]. Recent synthetic-biology studies have reconstructed TA biosynthetic pathways in yeast and achieved heterologous production of hyoscyamine and scopolamine; however, yields of 30–80 μg·L^−1^ remain below commercially viable levels [5]. Therefore, identifying alternative medicinal plants with adequate biomass, alkaloid content, and cultivation potential remains important for alleviating TA supply constraints.

**Figure 1:**
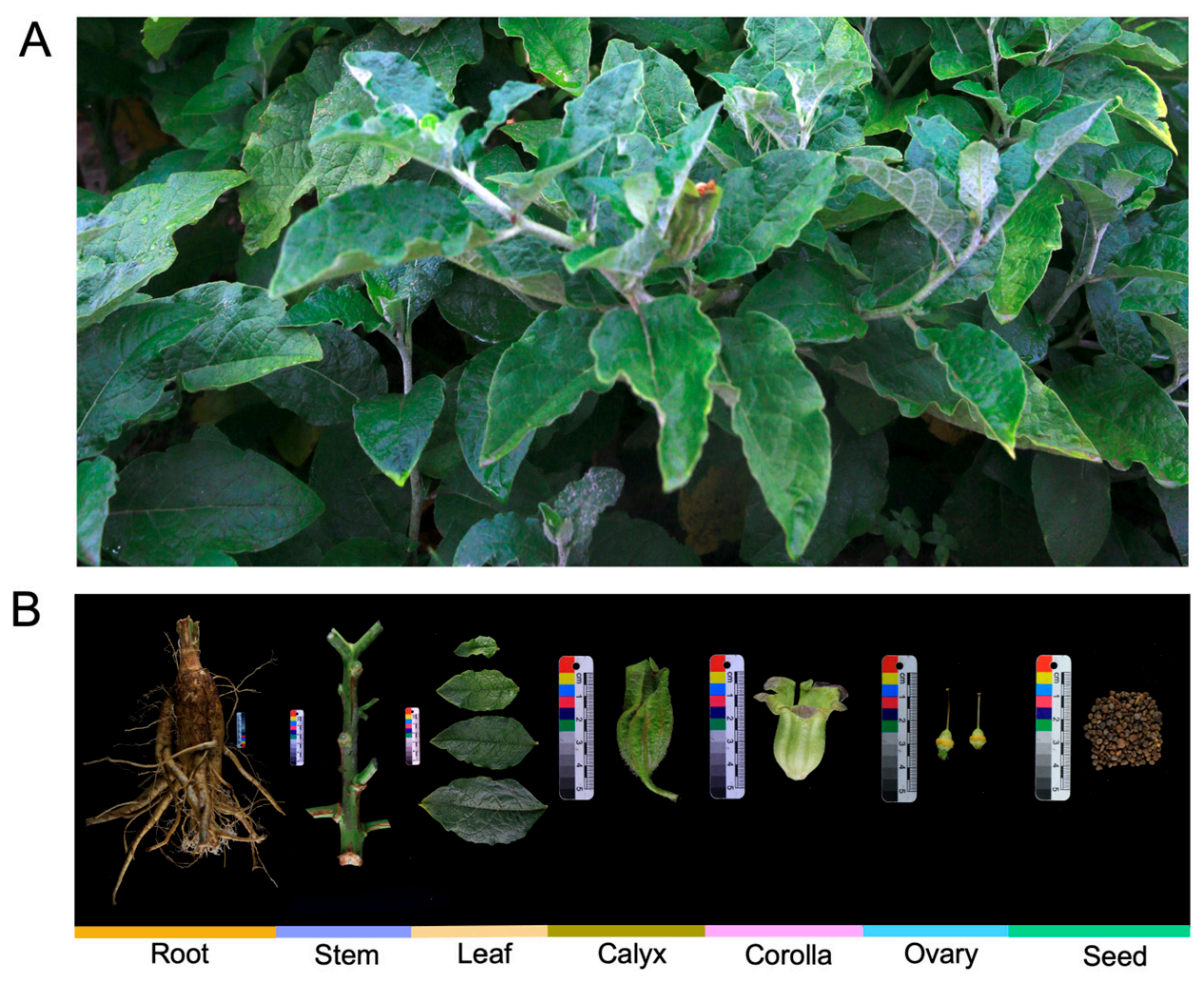

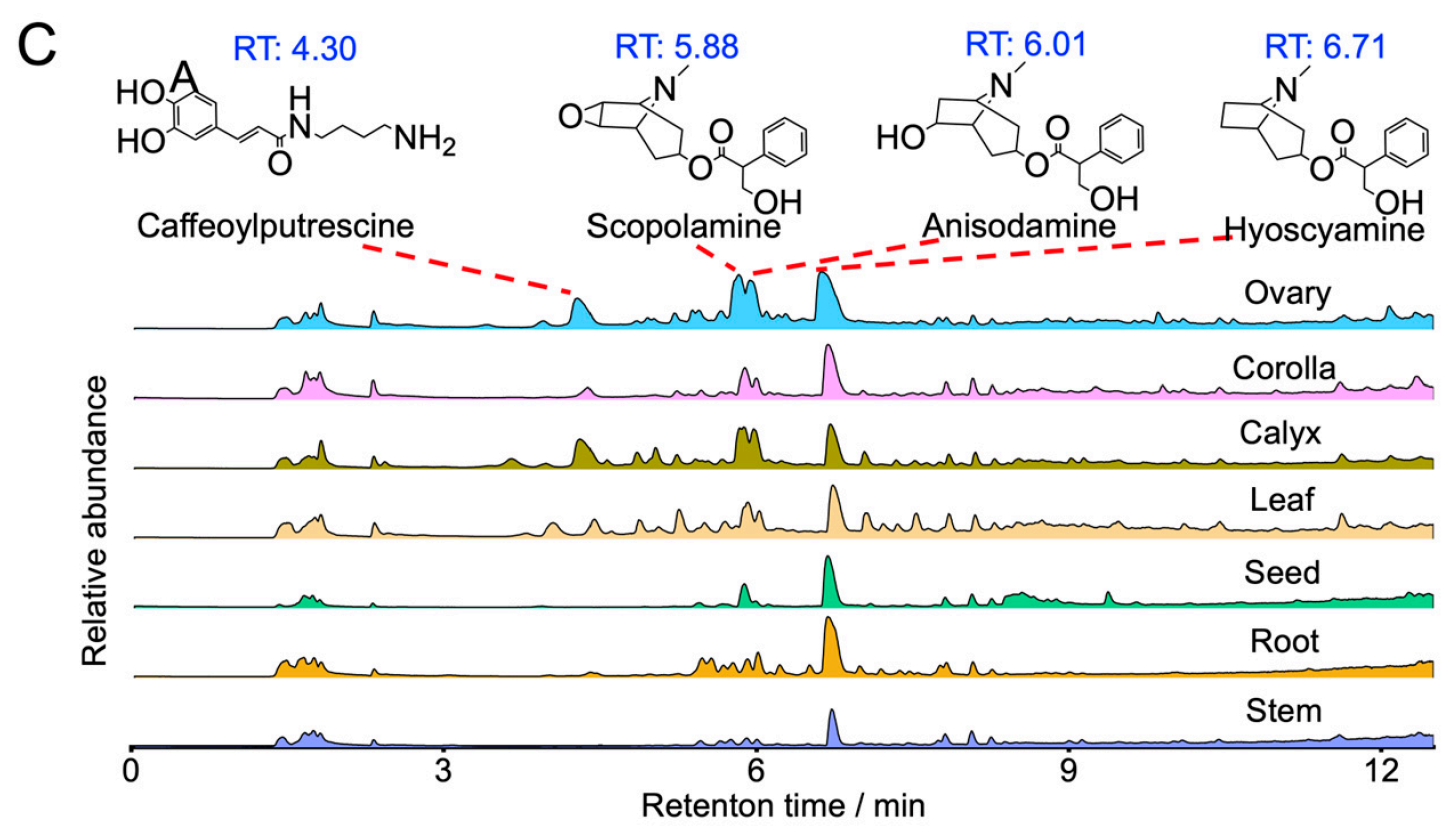
Plant morphology and total ion current (TIC) chromatograms of *Anisodus luridus*. (**A**) General appearance of *A. luridus*. (**B**) Sampled tissues, from left to right: root, stem, leaf, calyx, corolla, ovary, and seed; rulers provide size references. (**C**) UPLC-HRMS TIC chromatograms acquired in positive electrospray ionization mode, with tissue identities indicated by labels and colors. The horizontal axis shows retention time (min), and the vertical axis shows relative ion abundance. Red dashed lines link selected peaks to their assigned structures; retention times (RT) are given in minutes. Hyoscyamine and scopolamine were identified using authentic standards, whereas caffeoylputrescine and anisodamine were putatively annotated.

The genus *Anisodus* (Solanaceae) is a TA-rich taxon endemic to the Pan-Himalayan region [6]. Within this genus, *A. tanguticus* and *A. luridus* (Figure 1A) are collectively known as “Tangchong Nabao” in traditional Xizang medicine. Their medicinal use dates to the *Yuewang Yaozhen* and is further described in the *Jingzhu Bencao*, which classifies Tangchong as black, white, and variegated types. Traditionally, roots and seeds have been used to treat parasitic infections, mania, yellow water disease, and anthrax [6]. Compared with the relatively well-studied and widely distributed *A. tanguticus* [7], *A. luridus* remains comparatively underexplored as a medicinal resource in southeastern Xizang. Since 2016, studies of alkaloid content, gene function, and extraction-process optimization have provided preliminary support for its development potential. Zeng et al. (2016) used high-performance liquid chromatography (HPLC) to quantify alkaloids in *A. luridus* tissues and reported maximum hyoscyamine and scopolamine contents of 18.93 and 3.14 mg·g^−1^ dry weight (DW), respectively, in ovaries; both values exceeded those reported for *A. belladonna* [8]. Zhao et al. (2017) reported analogous TA biosynthetic pathways in *A. luridus* and *A. belladonna* [9]. Zhang et al. (2025) further optimized extraction and reported a TA yield of 6.211 mg·g^−1^ in roots [10]. Given the reported low scopolamine content of *A. belladonna* [3] and scopolamine’s approximately tenfold greater central activity than atropine [11,12], *A. luridus* represents a promising complementary industrial source. Its abundant biomass and elevated scopolamine content support further evaluation as a supplement to *A. belladonna* [8,10].

The medicinal value of *Anisodus* species may extend beyond TAs because phenylpropanoid polyamine alkaloids (PPAs) may contribute to biological activity. Long et al. (2014) isolated 11 amide alkaloids from the roots of *A. tanguticus*, demonstrating the chemical diversity of PPAs within the genus [13]. Wang et al. (2020) subsequently isolated scocycamides A and B, macrocyclic dicaffeoylspermidines with butyrylcholinesterase-inhibitory and antioxidant activities, suggesting potential relevance to Alzheimer’s disease [14]. PPAs occur in solanaceous plants, including potato and tomato [15]. Goji berry (*Lycium barbarum*) provides an illustrative example: Zhou et al. (2016) isolated 15 dicaffeoylspermidine derivatives (lycibarbarspermidines A–O) with reported neuroprotective and antioxidant activities [16]. Subsequent studies identified four additional derivatives (P–S), indicating that these compounds are prominent constituents of goji berry [17]. Five *L. barbarum* uridine diphosphate-dependent glycosyltransferases (LbUGT1–5) were reported to drive structural diversification through regioselective glycosylation [18]. Metabolomic profiling of 248 sample batches annotated 526 metabolites and indicated that glycosylated lycibarbarspermidines are predominant constituents in *L. barbarum* [19]. Kukoamine A has been reported to exert neuroprotective effects in neurotoxin-induced Parkinson’s disease models [20]. Collectively, these findings provide a rationale for profiling PPA candidates in *A. luridus* and related *Anisodus* species.

Although TAs and PPAs may co-occur in *A. luridus*, current research has largely focused on the targeted quantification of a limited subset of compounds and lacks systematic profiling of chemical constituents across multiple tissues. Traditional natural-product studies rely heavily on silica-gel column chromatography or preparative HPLC coupled with nuclear magnetic resonance (NMR) for structural elucidation. The isolation and identification of a single TA intermediate can require 2–3 months, which limits high-throughput analysis of large sample sets [21]. In contrast, ultra-performance liquid chromatography–high-resolution mass spectrometry (UPLC-HRMS) offers high analytical throughput and mass accuracy, enabling the simultaneous detection of numerous metabolic features [22]. However, conventional metabolomic analyses face two major bottlenecks: dependence on commercial spectral libraries and authentic standards leaves many features unannotated [23], and the absence of biologically informed prioritization can make it difficult to identify compounds of interest in large datasets [24]. Incorporating biosynthetic logic into data analysis may help address these limitations. Recent strategies, including isotopologue similarity networking (IsoNet) and theoretical chemical space mapping (CSM), integrate prior biochemical knowledge into metabolite annotation [19,25]. In non-model medicinal plants, established biosynthetic pathways can be used as a framework for putative annotation based on predicted enzyme-catalyzed modifications, such as retention-time shifts caused by hydroxylation or glycosylation, and characteristic tandem mass spectrometry (MS/MS) fragmentation patterns [26]. Because secondary-metabolite profiles can vary among tissues and with environmental conditions, an analytical framework that integrates biosynthetic rules with multi-tissue metabolomic profiles is important for characterizing the chemical profile of *A. luridus* and comparing it with established sources, such as *A. belladonna*.

Accordingly, we selected *A. luridus* to investigate alkaloid profiles across multiple tissues using a biosynthesis-informed putative annotation workflow. The workflow used TA and PPA biosynthetic pathways as a theoretical framework and applied substrate–product structural relationships, predicted enzyme-catalyzed modifications, and diagnostic MS/MS fragmentation patterns to annotate metabolites across seven sampled tissues (root, stem, leaf, calyx, corolla, ovary, and seed) (Figure 1B). We then used relative peak-area maps to examine the tissue-associated distributions of TA and PPA candidates. In this manuscript, “biosynthetic rules” refer to expected enzyme-catalyzed transformations and their predicted effects on retention time and MS/MS fragmentation. The results provide a basis for comparative studies with *A. belladonna* and for future evaluation of cultivation, tissue-selective harvesting, and extraction strategies.

## 2. Materials and Methods

### 2.1. Plant Materials

Roots, stems, leaves, calyces, corollas, and ovaries were collected simultaneously from healthy, six-year-old cultivated *A. luridus* plants at full bloom (July) at a cultivation site in Linzhi, Xizang, China (94°21′45.97″ E, 29°38′45.37″ N; approximately 3005 m above sea level). The cultivated *A. luridus* plants were morphologically identified by Prof. Xiaozhong Lan from Xizang Agriculture and Animal Husbandry University. For each tissue, materials from 15 individual plants were randomly assigned to five groups of three plants each; materials within each group were pooled to constitute one biological replicate (*n* = 5). Seeds were collected at maturity (September) from a distinct cohort of 15 individual plants grown at the same site. Samples were immediately frozen in liquid nitrogen, stored at −80 °C, freeze-dried, and ground to a fine powder.

### 2.2. Chemicals and Reagents

Ultrapure water was prepared using a Milli-Q water purification system (Merck Millipore, Burlington, MA, USA). LC-MS grade methanol, acetonitrile, and formic acid were purchased from Thermo Fisher Scientific (Waltham, MA, USA). Reference standards of hyoscyamine and scopolamine were obtained from Aladdin Biochemical Technology Co., Ltd. (Shanghai, China).

### 2.3. Metabolomics Detection

Briefly, 20 mg of freeze-dried, finely ground tissue was mixed with 1 mL of 70% (*v*/*v*) aqueous methanol (solid-to-liquid ratio: 20 mg·mL^−1^). Samples were vortex-mixed for 30 s at 2500 rpm and then sonicated in an ice bath for 30 min. The extracts were centrifuged at 14,000 rpm for 15 min at 4 °C. The supernatants were collected and passed through 0.22 μm polytetrafluoroethylene (PTFE) membrane filters compatible with organic solvents (Merck Millipore, Burlington, MA, USA) into LC-MS injection vials. A pooled quality-control (QC) sample was prepared by combining equal aliquots (15 μL) from each individual sample extract to monitor system stability. Metabolite extracts were analyzed using a UPLC-HRMS system comprising an Acquity H-Class UPLC instrument (Waters, Milford, MA, USA) coupled to a Xevo G2-XS quadrupole time-of-flight (Q-TOF) mass spectrometer (Waters, Milford, MA, USA). Chromatographic separation was performed on a BEH C18 column (2.1 × 50 mm, 1.7 μm; Waters, Milford, MA, USA). The detailed UPLC-HRMS parameters are summarized in Table 1.

### 2.4. Metabolomics Data Processing

Raw mass-spectrometry data were processed using Progenesis QI (https://www.nonlinear.com/progenesis/qi, accessed on 8 September 2026) for peak picking, alignment, baseline correction, and normalization to generate a normalized peak-area matrix. Preliminary putative annotations were obtained by searching public plant-metabolite databases, including MONA (https://mona.fiehnlab.ucdavis.edu, accessed on 8 September 2026) and PubChem (https://pubchem.ncbi.nlm.nih.gov, accessed on 8 September 2026). OPLS-DA was performed using SIMCA-P (version 13; Umetrics, Sweden), and heat maps were generated using TBtools-II (version 2.423) [27]. Statistical analyses were performed using SPSS (version 26.0; IBM Corp., Armonk, NY, USA). Normalized peak areas were compared across tissues using one-way analysis of variance (ANOVA) followed by Duncan’s multiple range test. Tissue groups that do not share a lowercase letter on the heat map differ significantly (*p* < 0.05). Metabolite identification and annotation confidence were reported according to the Metabolomics Standards Initiative (MSI) reporting criteria [28]. Hyoscyamine and scopolamine were identified at MSI Level 1 because their retention times and MS/MS spectra matched those of authentic standards analyzed under identical conditions. The remaining features were assigned to MSI Level 2 (putatively annotated compounds) or Level 3 (putatively characterized compound classes), as appropriate, based on MS/MS spectral evidence, public-database matches, and biosynthetic rationale. For the present dataset, “identified” refers to compounds confirmed with authentic standards, whereas “putatively annotated” refers to features assigned at MSI Levels 2 or 3; “annotated alkaloids” collectively refers to the 18 features used for comparative profiling.

## 3. Results

### 3.1. Total Ion Current (TIC) Profiles Across Tissues of A. luridus

Total ion current (TIC) chromatograms were acquired by UPLC-HRMS for seven sampled tissues of *A. luridus* (Figure 1C). The TIC profiles differed among tissues in peak number, intensity, and retention-time distribution. The ovary, corolla, calyx, and leaf showed broadly similar profiles, with numerous peaks between 3 and 7 min, whereas the other tissues had fewer detected peaks. Signals assigned to PPAs occurred predominantly between approximately 4 and 6 min, whereas signals assigned to TAs were more prominent between approximately 5 and 7 min. Signals corresponding to scopolamine (retention time (RT) = 5.88 min), anisodamine (RT = 6.01 min), and hyoscyamine (RT = 6.71 min) were detected in all tissues, although their relative peak areas differed. The signal assigned to caffeoylputrescine (RT = 4.30 min) had a higher relative peak area in the ovary, corolla, calyx, and leaf. These results showed tissue-associated variation in relative alkaloid abundance and motivated subsequent analyses.

### 3.2. Putative Annotation of TAs and PPAs Using Biosynthetic Rules

**Figure 2:**
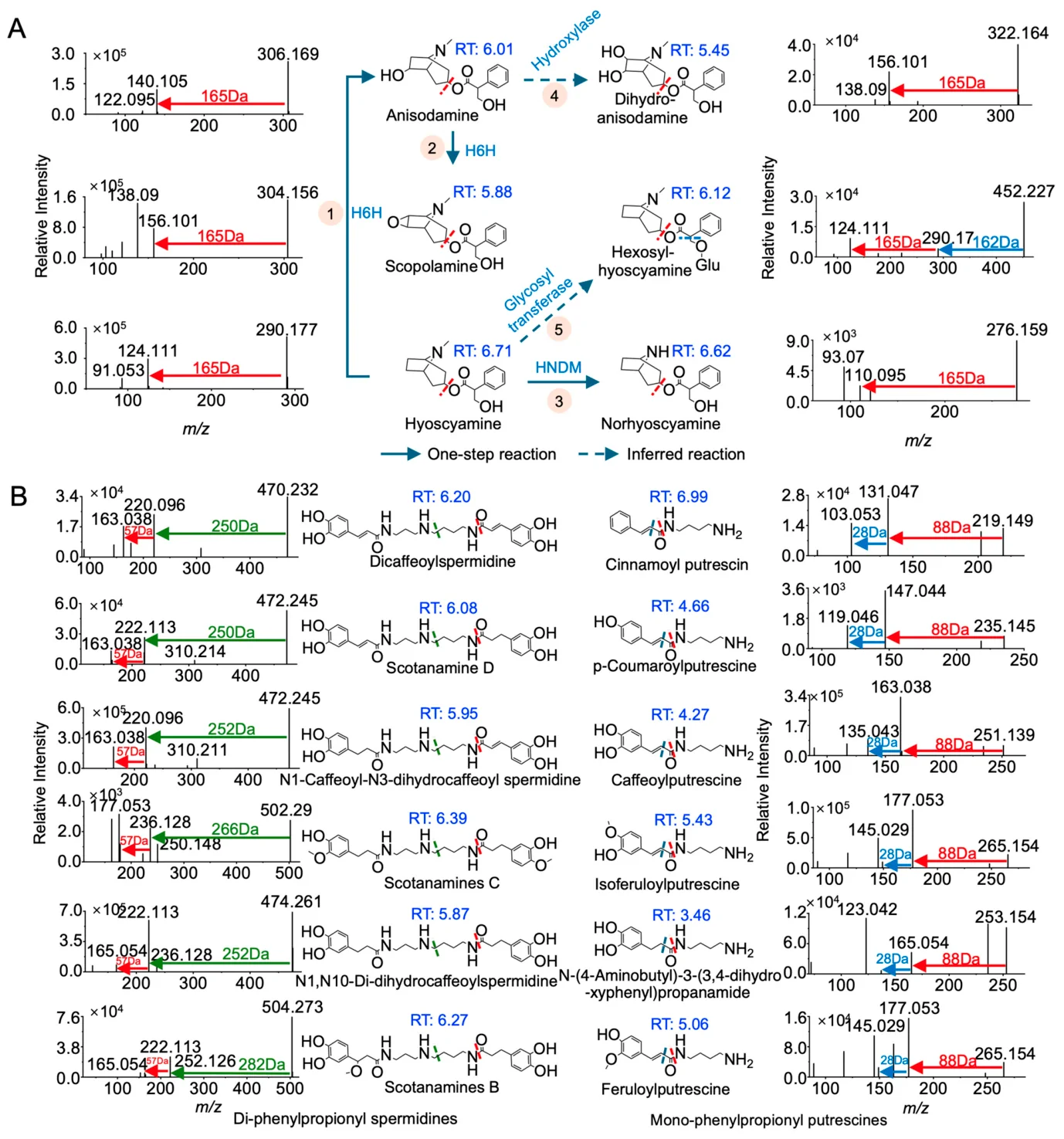
Biosynthesis-informed putative annotation of tropane alkaloids (TAs) and phenylpropanoid polyamine alkaloids (PPAs) using retention times and tandem mass spectrometry (MS/MS) fragmentation. (**A**) Six TA features and their proposed biosynthetic relationships. Numbers 1–5 denote hydroxylation, epoxidation, N-demethylation, additional hydroxylation, and glycosylation, respectively. Solid blue arrows indicate one-step reactions, and dashed blue arrows indicate inferred reactions. H6H, hyoscyamine 6β-hydroxylase; HNDM, hyoscyamine N-demethylase [1,29]. (**B**) Six di-phenylpropanoid spermidine candidates (left) and six mono-phenylpropanoid putrescine candidates (right). Colored dashed bonds and corresponding arrows indicate proposed fragmentation sites and mass differences (Da). RT, retention time (min); *m*/*z*, mass-to-charge ratio; Glu denotes the sugar moiety drawn.

Previous cloning and functional-characterization studies of TA biosynthetic genes in *A. belladonna* [5] and *A. luridus* [29] delineated enzyme-catalyzed modifications, including hydroxylation and glycosylation. We applied these biosynthetic rules—expected enzymatic transformations and their predicted effects on physicochemical properties and MS/MS fragmentation—to guide putative annotation. As shown in Figure 2A, six TA features were characterized using hyoscyamine, the core pathway product, as a reference. Hyoscyamine and scopolamine were identified using authentic standards (MSI Level 1), whereas the other four TA features were putatively annotated. Hyoscyamine, the most hydrophobic reference compound in this set, had the longest retention time (RT = 6.71 min). In its MS/MS spectrum, loss of the phenyllactic acid side chain (neutral loss of 165 Da) produced a characteristic tropine-ring fragment at mass-to-charge ratio (*m*/*z*) 124.111. Consistent with an additional hydroxyl group, anisodamine eluted earlier (RT = 6.01 min) and produced a hydroxyl-containing tropine-ring fragment at *m*/*z* 140.105. Dihydroanisodamine was putatively annotated in *A. luridus* for the first time and had an RT of 5.45 min; its fragment at *m*/*z* 156.101 was consistent with a dihydroxytropine-ring structure. Scopolamine, which contains an epoxide group, had an intermediate RT (5.88 min) between those of anisodamine and dihydroanisodamine; a fragment at *m*/*z* 156.101 supported an epoxide-containing tropine-ring assignment. Norhyoscyamine, putatively annotated as a demethylated hyoscyamine derivative, had a slightly shorter RT (6.62 min) than hyoscyamine. A hexosylhyoscyamine [30] feature was also putatively annotated in *A. luridus* for the first time. Its shorter RT (6.12 min) and sequential neutral losses of 162 Da, consistent with a hexose moiety, and 165 Da, yielding the tropine-ring fragment at *m*/*z* 124.111, were consistent with the putative annotation. Together, these observations suggested structural diversification of TAs in *A. luridus* through enzymatic modification. Several putative isomeric features were observed but were not discussed further because of low abundance or overlapping retention times.

PPA candidates are biosynthetically derived from putrescine and spermidine. BAHD-family acyltransferases can introduce phenylpropanoid groups, including cinnamoyl, p-coumaroyl, caffeoyl, and feruloyl moieties [31]. As shown in Figure 2B, the putatively annotated mono-phenylpropanoid putrescines exhibited an inverse relationship between retention time and the number of hydroxyl groups on the acyl side chain: retention times decreased from 6.99 min for cinnamoyl (no hydroxyl group) to 4.66 min for p-coumaroyl (one hydroxyl group) and 4.27 min for caffeoyl (two hydroxyl groups). The corresponding characteristic acyl fragments occurred at *m*/*z* 131.047, 147.044, and 163.038. Dihydrocaffeoylputrescine had the shortest observed retention time (3.46 min) and produced a characteristic fragment at *m*/*z* 165.054. Di-phenylpropanoid spermidines, including the scotanamine series, were putatively annotated using stepwise fragmentation patterns involving loss of an acylputrescine fragment followed by a 57 Da neutral loss. This pattern supported putative differentiation among candidate positional isomers. For example, dicaffeoylspermidine (RT = 6.20 min) produced fragment ions at *m*/*z* 220.096 and 163.038, both consistent with caffeoyl groups. Scotanamine D (RT = 6.08 min) produced fragments at *m*/*z* 222.113, consistent with a dihydrocaffeoyl moiety, and 310.214, consistent with a caffeoylspermidine fragment, supporting a putative hybrid structure. Similarly, candidates such as N^1^-caffeoyl-N^3^-dihydrocaffeoylspermidine (RT = 5.95 min) were putatively annotated based on characteristic fragment combinations.

### 3.3. Mapping of 18 Alkaloids in the Metabolic Network

**Figure 3:**
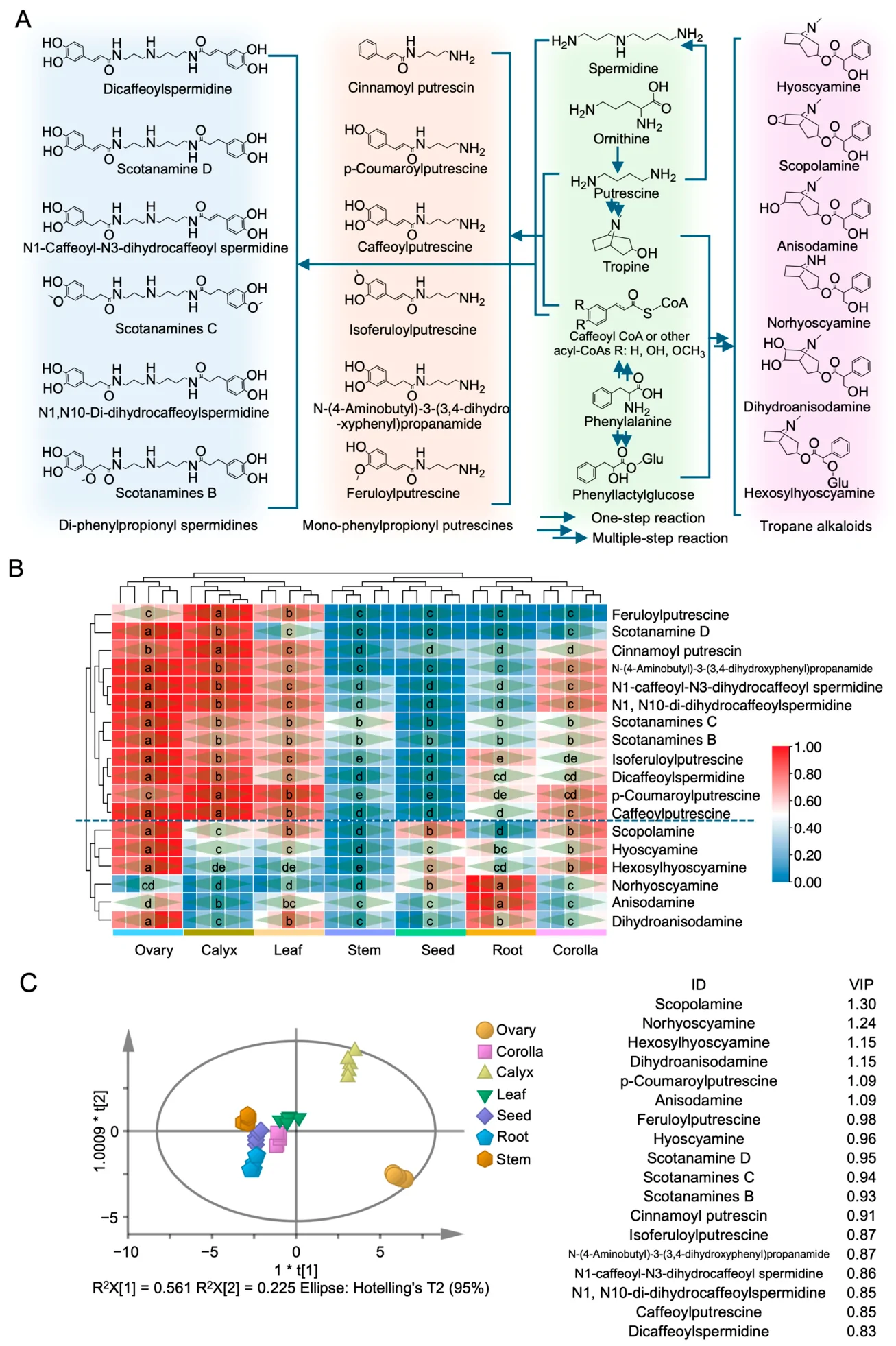
Proposed biosynthetic relationships and tissue-associated alkaloid profiles in *Anisodus luridus*. (**A**) Network linking ornithine and phenylalanine to tropane alkaloids (TAs), mono-phenylpropanoid putrescines, and di-phenylpropanoid spermidines. Single and paired arrowheads denote one-step and multistep relationships, respectively. CoA, coenzyme A; R, the indicated H, OH, or OCH_3_ substituent; Glu, the sugar moiety drawn. The network summarizes proposed relationships rather than measured metabolic fluxes. (**B**) Heat map of 18 annotated alkaloid features across seven tissues (*n* = 5 biological replicates per tissue). Rows represent alkaloid features and columns represent samples; dendrograms show hierarchical clustering. Colors show relative peak-area patterns on the displayed 0–1 scale (blue, lower; red, higher), rather than absolute concentrations. The horizontal dashed line separates PPA and TA clusters. For each alkaloid, tissue groups that do not share a lowercase letter differ significantly (one-way ANOVA followed by Duncan’s multiple range test, *p* < 0.05). (**C**) Orthogonal partial least squares discriminant analysis (OPLS-DA) score plot and variable importance in projection (VIP) values. Each point represents one biological replicate; colors and symbols denote tissues. The ellipse is the 95% Hotelling’s T^2^ boundary. The first two components explain 78.6% of the X variance (R^2^X [1] = 0.561; R^2^X [2] = 0.225); VIP > 1 was used to select variables contributing to tissue separation.

Mapping the 18 alkaloid annotations onto a network rooted in ornithine and phenylalanine (Figure 3A) was consistent with branch specialization from the shared intermediate putrescine. The annotations spanned three proposed branches: (i) a tropane alkaloid branch in which putrescine N-methyltransferase (PMT) forms N-methylputrescine, followed by oxidation and cyclization to generate the tropane scaffold and esterification with a phenylalanine-derived acid moiety [29]; (ii) a mono-phenylpropanoid putrescine branch generated by acylation with caffeoyl-coenzyme A (caffeoyl-CoA) or related acyl-CoAs through BAHD-family acyltransferases [31]; and (iii) a di-phenylpropanoid spermidine branch formed after conversion of putrescine to spermidine and subsequent acylation [32,33]. This mapping was consistent with the coexistence of these proposed branches in *A. luridus* and provided a rationale for future comparative studies with *A. belladonna*.

### 3.4. Tissue-Specific Accumulation Patterns of 18 Alkaloids

To examine the relative distribution of the annotated alkaloids, we analyzed normalized peak-area data across the seven sampled tissues. The heat map showed two broad clusters (Figure 3B). Cluster 1 contained TA features, including hyoscyamine, anisodamine, and hexosylhyoscyamine, and had higher relative peak areas in the ovary and root than in the other tissues. Cluster 2 contained PPA features, including caffeoylputrescine, dicaffeoylspermidine, and scotanamine-related compounds, and had higher relative peak areas in the calyx, leaf, and corolla, with lower values in the root and seed. Seed samples clustered with stem samples, suggesting a profile with moderate TA and low PPA relative abundances. This pattern should be interpreted cautiously because seeds were collected at maturity in September, whereas the other tissues were collected at full bloom in July.

### 3.5. OPLS-DA Model Screening of Biosynthesis-Related Differential Alkaloids

An OPLS-DA model was fitted using the peak areas of the 18 alkaloids as variables [34] (Figure 3C). The first two components explained 78.6% of the X variance (R^2^X [1] = 0.561; R^2^X [2] = 0.225). All samples fell within the 95% Hotelling’s T^2^ ellipse. In the score plot, samples from the same tissue grouped closely, whereas samples from different tissues showed separation: calyx and ovary samples occupied the right side of the plot, whereas stem and root samples were located on the left. Variable importance in projection (VIP) analysis, using a threshold of >1, ranked scopolamine (VIP = 1.30) and norhyoscyamine (VIP = 1.24) as the principal variables associated with separation among the sampled tissues. These findings suggested that variation in TA-associated signals contributed substantially to differences among the sampled tissue profiles.

## 4. Discussion

### 4.1. Biosynthetic Logic as a Guide for Putative Annotation in Non-Model Plant Metabolomics

Untargeted liquid chromatography–high-resolution mass spectrometry (LC-HRMS) studies of non-model medicinal plants often contain a large proportion of unannotated features due to the limited availability of authentic standards and curated spectral libraries [23]. To address this limitation, metabolite annotation approaches increasingly integrate biosynthetic knowledge with data-driven analysis. Molecular networking platforms, such as Global Natural Products Social Molecular Networking (GNPS), facilitate spectral-similarity-based clustering and propagation of annotations [26]. Other frameworks, including Metabolic Reaction Network-based Recursive Metabolite Annotation (MetDNA), the Knowledge-Guided Multi-layer Network (KGMN; i.e., MetDNA2), MetDNA3, and Network-Based Inference for Metabolite Identification (NetID), use known metabolites and metabolic reaction or network relationships to support the recursive putative annotation of unknown features [35,36,37,38]. A recent CSM study reported the identification of over 100 PPA analogs in goji berry (*Lycium barbarum*) through biosynthetic-rule-guided exploration [19]. Collectively, these approaches employ biosynthetic logic to link MS data with candidate structures, thereby reducing annotation uncertainty. The workflow used here was conceptually informed by these approaches and focused on two characteristic Solanaceae metabolite classes, TAs and PPAs. We combined expected enzyme-catalyzed transformations with diagnostic retention-time and MS/MS fragmentation patterns to support putative annotations. For example, a hexosylhyoscyamine feature was putatively annotated on the basis of its earlier retention time and a 162 Da neutral loss consistent with a hexose moiety [30]. Similarly, an 88 Da neutral loss diagnostic of the putrescine backbone supported the annotation of mono- and disubstituted PPA candidates [31]. This workflow may facilitate class-focused putative annotation in non-model solanaceous medicinal plants when taxon-specific spectral resources are limited.

### 4.2. Reevaluating the Medicinal Value of A. luridus: Beyond a TA-Centric Framework

Previous studies of *A. luridus* have primarily emphasized TA content and extraction protocols [8,10], resulting in a root-focused quality-evaluation approach. Qiang et al. (2014) reported that TA biosynthetic genes in *A. belladonna* were expressed predominantly in lateral roots and that synthesized TAs were translocated and deposited in aerial tissues [39]. Wang et al. (2005) found that TA contents in the roots of cultivated and wild *Anisodus tanguticus* were higher than those in aerial tissues [40], which may help explain the traditional emphasis on root harvesting. Using the biosynthesis-informed workflow, we identified hyoscyamine and scopolamine and putatively annotated additional TA and PPA candidates in *A. luridus*, indicating a more diverse alkaloid profile than previously described. The two classes showed distinct relative distribution patterns: TA-associated signals were higher in roots and ovaries, whereas PPA-associated signals were higher in leaves and floral tissues. The higher relative abundance of TAs in roots and ovaries is consistent with, but does not test, predictions derived from optimal-defense theory [41]. Conjugated double bonds in PPA phenylpropanoid moieties may contribute to antioxidant or photoprotective potential [42]. This pattern is compatible with possible functional partitioning of these metabolite classes; however, the present data do not establish their ecological or physiological functions. The proposed links between PPAs, antioxidant or photoprotective potential, and high-altitude stress responses therefore require direct validation in controlled experiments. Studies of goji berry (*L. barbarum*) have reported neuroprotective activity for some structurally related metabolites [16,43,44]. These observations justify future bioactivity testing of the PPA candidates detected in *A. luridus*, but they do not establish comparable activity in this species. Cross-species evidence indicates that UV exposure can induce phenylamide accumulation in rice leaves [45], providing indirect context for hypothesized ecological roles of PPAs in *A. luridus*. The observed relative enrichment of PPA-associated signals in aerial tissues may inform future studies of tissue-selective harvesting and extraction; its practical value should be assessed after targeted quantification and bioactivity validation.

### 4.3. Limitations and Future Perspectives

Several limitations should be acknowledged. First, because authentic standards, internal standards, and calibration curves were unavailable for most analytes, the peak-area data provide semi-quantitative relative abundances rather than absolute concentrations [46]. Future studies should use quantitative NMR (qNMR) or stable-isotope-labeled internal standards to enable absolute quantification. Second, the pooling strategy produced sufficient material to detect low-abundance metabolites but did not permit assessment of inter-individual variation. Individual plants should therefore be analyzed separately in future work. Third, seeds were collected at physiological maturity in September, whereas vegetative and floral tissues were sampled at full bloom in July. Because secondary-metabolite profiles vary across development [1], the observed seed profile may reflect both tissue and developmental-stage effects. Finally, the proposed neuroprotective and ecophysiological roles of the PPA candidates are based on structural similarity and cross-species evidence rather than direct testing of *A. luridus* extracts. These hypotheses should be evaluated through bioactivity-guided fractionation, in vitro and in vivo assays, and dedicated stress-physiology experiments. Accordingly, future integration of multi-omics network reconstruction with bioactivity-guided fractionation could provide a systems-level framework for further evaluating *A. luridus* as a medicinal resource.

## 5. Conclusions

In this study, we applied a biosynthesis-informed metabolomics workflow to characterize alkaloid features in *A. luridus*. Eighteen alkaloid features, including 6 TAs and 12 PPAs, were characterized: hyoscyamine and scopolamine were identified using authentic standards, and the remaining 16 features were putatively annotated. Their relative peak areas differed across the sampled tissues. The observed distribution patterns provide a basis for future comparisons with *A. belladonna* and for studies of cultivation, harvesting, extraction, and metabolic engineering. In practice, the relative enrichment of TA-associated signals in roots and ovaries and PPA-associated signals in leaves and floral tissues can guide the selection of tissues for cultivation trials and selective harvesting. Targeted quantification across developmental stages, together with measurements of tissue biomass and plant regrowth, is needed to determine harvest timing and assess alkaloid yield and the feasibility of repeated harvesting. Integrated multi-omics and targeted validation will be needed to define the regulatory basis and practical value of these patterns.

## Figures and Tables

**Table 1:** UPLC-HRMS analytical parameters for metabolomic profiling of *A. luridus* tissues.

Category	Parameter	Setting
UPLC conditions	column temperature	40 °C
	flow rate	0.3 mL·min^−1^
	injection volume	5 μL
	mobile phase A	0.1% formic acid in water
	mobile phase B	0.1% formic acid in acetonitrile
	gradient program	0–5 min, 95 → 70% A; 5–10 min, 70 → 5% A; 10–13 min, 5% A; 13–17 min, 95% A
HRMS conditions	ionization mode	positive ESI (ESI^+^)
	capillary voltage	3 kV
	cone voltage	40 V
	cone gas flow	50 L·h^−1^
	desolvation gas flow	800 L·h^−1^
	source temperature	100 °C
	desolvation temperature	350 °C
	low collision energy	6 eV
	high collision energy	30–60 eV (ramped)
	scan mode	centroid, 0.1 s scan time
	acquisition mode	MS^E^
	lock-mass calibrant	leucine enkephalin (200 pg·μL^−1^) (Waters, Milford, MA, USA)
	mass range	50–1200 *m*/*z*

## Data Availability

The datasets generated during and/or analyzed during the current study are available from the corresponding author on reasonable request.
